# Effect of the Lights4Violence intervention on the sexism of adolescents in European countries

**DOI:** 10.1186/s12889-022-12925-3

**Published:** 2022-03-19

**Authors:** Belén Sanz-Barbero, Alba Ayala, Francesca Ieracitano, Carmen Rodríguez-Blázquez, Nicola Bowes, Karen De Claire, Veronica Mocanu, Dana-Teodora Anton-Paduraru, Miriam Sánchez-SanSegundo, Natalia Albaladejo-Blázquez, Ana Sofia Antunes das Neves, Ana Sofia da Silva Queirós, Barbara Jankowiak, Katarzyna Waszyńska, Carmen Vives-Cases

**Affiliations:** 1 National School of Public Health, Carlos III Institute of Health, Avda. Monforte de Lemos, 5–28029 Madrid, Spain; 2 CIBER of Epidemiology and Public Health (CIBERESP), Madrid, Spain; 3 Research Network on Health Services for Chronic Diseases (REDISSEC), Madrid, Spain; 4 Department of Human Studies-Communication, Education and Psychology - LUMSA University of Rome, Rome, Italy; 5 National Center of Epidemiology, Carlos III Institute of Health, Madrid, Spain; 6 CIBER in Neurodegenerative Diseases (CIBERNED), Madrid, Spain; 7 Department of Applied Psychology, Cardiff Metropolitan University, Wales, UK; 8 Mother and Child Medicine Deparment, Gr.T. Popa University of Medicine and Pharmacy, Iasi, Romania; 9 Faculty of Health Science, University of Alicante, Alicante, Spain; 10 University of Maia and CIEG/ISCSP-ULisboa, Porto, Portugal; 11 University Institute of Maia – ISMAI, Maia, Portugal; 12 Faculty of Educational Studies, Adam Mickiewicz University, Poznań, Poland

**Keywords:** Ambivalent sexism, Intervention, Adolescents

## Abstract

**Background:**

Sexism results in a number of attitudes and behaviors that contribute to gender inequalities in social structure and interpersonal relationships. The objective of this study was to evaluate the effectiveness of Lights4Violence, an intervention program based on promoting health assets to reduce sexist attitudes in young European people.

**Methods:**

We carried out a quasi-experimental study in a non-probabilistic population of 1146 students, aged 12–17 years. The dependent variables were the difference in the wave 1 and wave 2 values in the subscales of the Ambivalent Sexism Inventory: benevolent sexism (BS) and hostile sexism (HS). The effect of the intervention was evaluated through linear regression analyses stratified by sex. The models were adjusted by baseline subscales scores, socio-demographic and psychological variables.

**Results:**

In girls, we observed a decrease in BS in the intervention group compared to the control group (β = − 0.101; *p* = 0.006). In the wave2,, BS decreased more in the intervention group compared to the control group in girls with mothers with a low level of education (β = − 0.338; *p* = 0.001), with a high level of social support (β = − 0.251; *p* < 0.001), with greater capacity for conflict resolution (β = − 0.201; *p* < 0.001) and lower levels of aggressiveness (β = − 0.232, *p* < 0.001). In boys, the mean levels of HS and BH decreased in wave 2 in both the control and intervention groups. The changes observed after the wave 2 were the same in the control group and in the intervention group. No significant differences were identified between both groups.

**Conclusions:**

The implementation of the Lights4Violence was associated with a significant reduction in BS in girls, which highlights the potential of interventions aimed at supporting the personal competencies and social support. It is necessary to reinforce the inclusion of educational contents that promote reflection among boys about the role of gender and the meaning of the attributes of masculinity.

**Trial registration:**

Clinicaltrials.gov : NCT03411564. Unique Protocol ID: 776905. Date registered: 26-01-2018.

## Background

Sexism has been defined in different ways over time and across cultures [1]. Traditionally, sexism towards women has been defined as a series of discriminatory behaviors based on values and attitudes that consider that the women is by nature inferior to the men [2]. This is defined by Glick as hostile sexism (HS) and represents a sexist antipathy toward women [3] who are perceived as competing with men when they behave in a “non traditional” way [4]. In recent decades, a more subtle dimension of sexism, benevolent sexism (BS) has emerged [5]. BS is expressed through protective attitudes towards women that are expressed socially as positive signs of courtesy and respect, but that place women on an inferior plane. In this dimension women are considered to be weaker and in need of protection, care and companionship by men. These two dimensions of sexism, HS and BS, have been found to be positively correlated to each other and comprise what is referred to as ambivalent sexism [1].

Sexism emphasizes gender roles and defines heterosexual romantic relationships, as the only way to feel complete and reach happiness. In this sense, sexual LGB orientation or transsexual and transgender people also experience discrimination due to sexist attitudes and behaviors [6]. Sexism, along with other axes of oppression, such as racism, class, disability, homophobia and transphobia, gives rise to multiple forms of discrimination [7]. Sexist attitudes, behaviors and values generate gender inequalities that are manifest in the social structure and in interpersonal relationships. There is currently a consolidated body of evidence that shows an association between sexist attitudes and risky sexual relationships among young people [8], emotional dependence [9], low levels of education among women [10] and poor quality of affective relationships [11]. In both sexes, HS predicted more favorable attitudes toward bullying [12] men and women, high score in BS and HS sexism were more likely to minimize DV, whereas those high only in BS were more likely to blame the victim [13], When men had a high relational dependence and perceived that women had a low in relationship commitment, HS was related with more aggressive toward their female partner [14]. From a health promotion perspective, encouraging non-sexist attitudes and personal abilities in the adolescent population is key to the development of positive, healthy and non-sexist interpersonal relationships as well as the prevention of dating violence [15].

The magnitude and the prevalence of adverse consequences of teen dating violence among young people - both short and long-term [16, 17] justify the need to identify prevention policies and interventions that promote their well-being. According to the European Violence against Women Survey [18], the prevalence of current physical and/or sexual intimate partner violence (IPV) among young women ages 18–29 is 6%, and it is 48% in the case of lifetime psychological IPV. In contrast, the registered prevalence among adult women over age 30 was around 4 and 32%, respectively. Even though studies involving boys are less frequent it has been observed that boys adolescents also experience dating violence victimization, but the dynamics and consequences may be more severe for girls [19].

Stereotyped attitudes about the protective role of men and women’s need for protection and care reflect fundamental components of benevolent sexism that are sometimes present from childhood [20]. Adolescence is a developmental stage that is especially relevant for questioning negative social models learned in infancy [21]. It is this developmental stage on which men and women define their identities and behavior styles in intimate relationships. Adolescence also represents, an opportunity to promote healthy relationships.

Nowadays, most dating prevention intervention programs whose results have been published in the scientific literature, have been carried out mainly in the United States [22]. A recent meta-analysis highlighted the importance of these interventions being integral, although the context where they are most effective is in the educational field [23]. These interventions have targeted both the general young population and youth at risk of suffering from or committing gender violence like children exposed to domestic violence, or witnesses of maternal abuse. Most interventions have been focused on increasing knowledge of violence and traditional gender roles [24], in addition to providing skills that allow young people to confront frustration and resolve conflicts in a non-violent way [22], and increasing awareness and skills about appropriate bystander interventions among adolescents [25, 26]. Although the evaluations of these interventions are heterogeneous, the results show that it is possible to reduce victimization and perpetration of dating violence [23, 27, 28].

This study was carried out in the context of the Lights4Violence Project, an educational intervention which aims to promote dating violence protective assets among secondary school students from different European cities (Alicante, Rome, Iasi, Matosinhos, Poznan and Cardiff) [29]. This project was based on the model for positive youth development, which emphasizes youth strengths, stressing the development of capacities (personal, moral, cognitive, conceptual and social) that support young people in resisting risk factors, and reducing or coping with problems such as violence perpetration or victimization [30]. We integrated the aim of supporting adolescents in challenging sexism due to the promising results previously observed in interventions that bring factors such as gender equality, violence acceptability, non-violent conflict resolution, and other healthy development skills to bear in preventing dating violence among young people [23].

In this study, we aimed to evaluate the effectiveness of the Lights4Violence project in decreasing sexism among secondary school students. The study is based on the hypothesis that the intervention implemented in the Lights4Violence project will produce a decrease in the mean values of sexism in boys and girls in the intervention group but not in the control group. Also, given that prior studies have shown that the existence of sexist attitudes among Spanish adolescents may differ between sex [31], with boys showing a higher degree of HS than girls, the analyses were stratified by sex.

## Methods

### Lights4Violence intervention

The intervention “Filming Together to See Ourselves in a New Present” –Lights4Violence- intervention aimed to provide adolescents with assets and skills that promote positive couple relationships. This intervention has as a framework the Positive Youth Development Model [32, 33]. The model integrates different areas of intervention for the promotion of positive adolescent development (personal development -such as empathy; cognitive -such as the ability to solve problems; emotional -such as aggressiveness; social -as the ability to solve problems or relate to others or social support; and, moral -such as sexism). We selected for the project those that were related with the promotion of positive intimate relationship or dating violence protective factors [34]. In this study, we selected covariates among our whole project variables that were also associated with sexism [35].

Lights4Violence project implemented an educational intervention in secondary schools by project members and/or teachers with prior training. The intervention consisted of two different parts. Students first participated in 10 theoretical and practical sessions for an average of 55 min per session. Later, during 5 practical sessions lasting 55–60 min, students filmed a series of video capsules put together into short films in which, using the knowledge and skills they had acquired, they resolved situations of conflict between couples. The intervention ended with a public showing of the films with the support of city councils and other public institutions. The intervention was carried out during the period October 2018 and April 2019. More information about the intervention can be found in the following article [29].

### Study design and sample

The study was a quasi-experimental study using a convenience sample of secondary students (age range: 12–17) in six European cities: Alicante (Spain), Mathosinos (Portugal), Cardiff (United Kingdom), Roma (Italy), Poznán (Poland) and Iasi (Romania). The intervention group included students from 12 school centers selected using viability criteria. The control group was made up of students from six schools (different from the intervention schools) that were located in the same cities where the intervention was carried out. Data collection was carried out through an online questionnaire, with an average duration of 45 min. The questionnaire was administered to both the case and control groups prior to the beginning of the intervention (wave 1), and approximately 6 months after the end of the intervention (wave 2). The project control group received no alternative intervention during this time period. A statistical power analysis was performed for sample size estimation (initial sample designed for 1300 students), based on data from a previous published random-effects meta-analysis of 23 studies about school-based interventions that aimed to prevent violence and negative attitudes in teen dating relationships [36]. The percentage of cases lost during follow-up was 25.7%. Given that the analysis was stratified by sex, 0.6% (*n* = 9) of cases was eliminated when people answered “other” to the question asking about their sex. Sample size was determined before any data analysis and it was not increased after analysis. The final sample used for this study included 1146 people. All the obtained measures are discussed in results section. We did not do any manipulations or exclusion results.

### Variables

The outcome variable was sexism measured with the Ambivalent Sexism Inventory (ASI) scale [5]. ASI includes two subscales: benevolent sexism (BS) and hostile sexism (HS). Each of the subscales is made up of 11 items scoring the level of agreement or disagreement in a likert-type scale with six categories. Higher scores on the scale indicate greater levels of sexism (range: 0–110).

Based on prior evidence [37] and the differences between cases and controls identified in our sample (Table 1), the following were included as covariables:

**Table 1:** Sample description for wave 1, Lights4Violence project

	Girls					Boys				
	Control (***n*** = 358)		Intervention (***n*** = 340)		p*	Control (***n*** = 213)		Intervention (***n*** = 235)		p*
	***n***	%	***n***	%		***n***	%	***n***	%	
Age					0.014					0.001
< =13 years	94	26.3	123	36.2		66	31.0	112	47.7	
14–15 years	186	52.0	159	46.8		111	52.1	95	40.4	
> 15 years	78	21.8	58	17.1		36	16.9	28	11.9	
Mother’s education					0.015					0.350
Primary and lower	34	9.5	51	15.0		26	12.2	40	17.0	
Secondary	147	41.1	156	45.9		77	36.2	77	32.8	
University	172	48.0	133	39.1		106	49.8	118	50.2	
Missing	5	1.4	0	0.0		4	1.9	0	0.0	
Dating violence experience					0.001					0.105
I have never been …	171	47.8	116	34.1		80	37.6	64	27.2	
Yes	67	18.7	69	20.3		36	16.9	41	17.4	
No	120	33.5	151	44.4		97	45.5	122	51.9	
Missing	0	0.0	4	1.2		0	0.0	8	3.4	
Student Social Support Scale					0.008					0.030
Low (< 232 points)	142	39.7	99	29.1		75	35.2	56	23.8	
Middle (232–276 points)	103	28.8	127	37.4		68	31.9	87	37.0	
High (> 276 points)	113	31.6	114	33.5		70	32.9	92	39.1	
Assertiveness					0.458					0.198
Low (< 82 points)	117	32.7	101	29.7		79	37.1	77	32.8	
Middle (82–92 points)	116	32.4	103	30.3		65	30.5	62	26.4	
High (> 92 points)	125	34.9	133	39.1		69	32.4	95	40.4	
Missing	0	0.0	3	0.9		0	0.0	1	0.4	
Social Problem-Solving Inventory Revised					0.493					0.041
Low (< 54 points)	119	33.2	109	32.1		73	34.3	66	28.1	
Middle (54–64 points)	115	32.1	97	28.5		80	37.6	75	31.9	
High (> 64 points)	124	34.6	130	38.2		60	28.2	92	39.1	
Missing	0	0.0	4	1.2		0	0.0	2	0.9	
Aggression Questionnaire Refined					0.208					0.255
Low (< 23 points)	131	36.6	103	30.3		58	27.2	80	34.0	
Middle (23–31 points)	109	30.4	119	35.0		64	30.0	66	28.1	
High (> 31 points)	118	33.0	113	33.2		91	42.7	87	37.0	
Missing	0	0.0	5	1.5		0	0.0	2	0.9	

*Chi-square test for differences by control and intervention group (not counting missing values)

#### Assets related to external support resources

- Perceived social support was evaluated using the Student Social Support Scale (SSSS) [38]. Students answered 60 questions in 6 likert-type categories that measure the level of social support in five areas: parents, teachers, classmates, close friends and people of the school. Example items include “My parents show they are proud of me”; “My teachers explain things that I don’t understand”; “My close friend spends time with me when I’m lonely”. A higher score indicates greater social support (range: 60–360). The variable was divided into tertiles: low social support (< 232 points), medium (232–276 points), high (> 276 points).

#### Assets related to personal competencies

- Assertiveness was measured using the Assertive Interpersonal Schema Questionnaire (AISQ) [39]. The scale contains 21 items in five likert-type response categories, ranging from completely false to completely true (range: 21–105). Example items are “I feel I am special to some people”; “I possess as many skills as most people”; “When I am sad, angry, or upset, I have someone to support me and help me feel”. The variable was categorized into tertiles: low assertiveness (< 82 points), medium (82–92 points), high (> 92 points)
- To evaluate the capacity of students to resolve social conflicts, we used the Social Problem-Solving Inventory-Revised Scale (SPSI-R) [40]. The scale consists of 25 items in categories with likert-type responses that range from 0 to 4 (range: 0–100). Example items: “I try to see my problems as challenges”; “When I solve problems, I try to predict the pros and cons of each option”. The capacity to resolve conflicts was categorized into tertiles: low capacity to resolve conflicts (< 54 points), medium (54–64 points) and high (> 64 points).
- Aggressiveness of the students was measured using the abbreviated version of the Aggression Questionnaire (AQR-12) [41, 42]. The questionnaire consists of 12 items in 5 categories with likert-type responses (range: 12–60). Example items include “Given enough provocation”; “I may hit another person”; “I can’t help getting into arguments when people disagree with me”. The variable was categorized into tertiles. High scores indicated greater aggressiveness: low (< 23 points), medium (23–31 points) and high (> 31 points).

### Other adjustment variables

#### Sociodemographic variables: sex, age, mother’s education level

- Variables related to relationships and inter-partner violence that include both control and fear, such as exposure to physical and sexual violence by a partner.
- Benevolent sexism or hostile sexism at baseline was including for controling its effects as possible moderator.

### Data analysis

#### Descriptive analysis

All the analyses were stratified by sex. First, we performed a descriptive study to calculate the differences in baseline between the control and intervention groups using a chi-square test. Then we analyzed the mean differences in BS and HS, between wave 1 (W1) and wave 2 (W2), in the control and intervention groups through paired t-tests for repeated measures. The magnitude of the mean difference over time was measured using the effect size coefficient, calculated using Cohen’s d. The effect size was considered small when Cohen’s d was around 0.2, medium when it was around 0.5 and large when it was around 0.8. Later, we analyzed differences in the mean values of BS and HS between W1 and W2 for each of the covariables, both in the control group and in the intervention group.

#### Intervention effect analysis

In order to analyze the intervention’s effects, we performed linear regression models. The outcome variable was the difference in the value obtained in the ASI subscales -BS and HS- between W1 and W2 (*Y*_*i*2_ − *Y*_*i*1_) where *Y*_*i*2_ is the observation for student in W2 and *Y*_*i*1_ is observation for student i in W1 (eq. 1). The intervention effect is identified by the variable group (control/intervention). Models were adjusted by the following covariates: outcome value in baseline (*Y*_*i*1_), country, age, mother’s education and dating violence, and the following scales measured in W1: SSSS, AISQ, SPSI-R, AQR-12, BS/HS.

1 $${\displaystyle \begin{array}{c}{Y}_{i2}-{Y}_{i1}={\beta}_0+{\beta}_1{Y}_{i1}+\dots +{\varepsilon}_i\\ {}{\beta}_0\ and\ {\beta}_1= coefficients\ of\ the\ model;{\varepsilon}_i= random\ errors\end{array}}$$

To analyze whether the intervention had a different effect for each of the categories of the covariables included in the model, we explored the interactions between the group variable (control vs. intervention) and each covariable. For those interactions that were significant we analyzed the differences by group (intervention and control) in the different categories of the covariables. All of the analyses were stratified by sex and used the software program Stata 14.0.

## Results

We collected 1555 questionnaires in W1 and 1434 questionnaires in W2, resulting in 1155 questionnaires matched using the student’s code (578 control and 577 intervention). Nine questionnaires were excluded because they had missing values for the sex variable. The final dataset used included 1146 questionnaires, 575 from the intervention group (59.1% girls) and 571 from the control group (62.7% girls). Table 1 describes the sociodemographic characteristics and the distribution in tertiles of the scales used in the baseline (W1) for each group (intervention and control), stratified by sex.

### Results on benevolent sexism

Table 2 shows the mean values of BS in the variables included in the analysis, in both periods –W1 and W2-, by group (control / intervention) and by sex. In W2, there was a significant reduction in BS in both groups (control/intervention) and among both sexes. The effect size was greater in the intervention group (effect size total: 0.33; effect size girls: 0.23; effect size boys: 0.18) than in the control group (effect size total: 0.11; effect size girls: 0.07; effect size boys: 0.09). Among the girls in the intervention group, in W2 there was a significant reduction in the mean values of BS with respect to W1 in all of the studied variables. Among the boys in the intervention group, there was a significant reduction in BS in the extreme categories of those variables that indicate higher family socioeconomic level (mother with university studies) (*p* < 0.001)], greater social support [SSSS high (*p* = 0.004)], and greater social competencies and skills [ASIQ high (0.029), SPSI-R high (*p* = 0.005) and AQR-12 low (*p* = 0.002)]). BS decreased in some of the categories of the control group in both sexes.

**Table 2:** Means and standard deviations for benevolent sexism by demographic, socioeconomic and violence-related Variables at baseline

	Girls						Boys					
	Control			Intervention			Control			Intervention		
	Pre-test	Post-test		Pre-test	Post-test		Pre-test	Post-test		Pre-test	Post-test	
	Mean (SD)	Mean (SD)		Mean (SD)	Mean (SD)		Mean (SD)	Mean (SD)		Mean (SD)	Mean (SD)	
Total	27.0 (10.5)	25.9 (11.0)	*	28.0 (11.7)	24.2 (11.7)	**	30.1 (10.4)	28.7 (10.5)	*	29.8 (10.0)	27.1 (11.4)	**
Age												
< =13 years	24.7 (10.3)	23.4 (11.1)	ns	25.7 (11.5)	20.6 (9.7)	**	30.3 (12.2)	28.6 (12.1)	ns	28.6 (9.3)	25.0 (11.0)	*
14–15 years	27.8 (10.4)	26.3 (11.0)	*	30.4 (10.8)	28.5 (11.1)	*	30.5 (9.3)	29.1 (9.0)	ns	31.6 (10.4)	30.7 (10.9)	ns
> 15 years	27.8 (10.4)	28.0 (10.5)	ns	25.9 (13.1)	19.8 (13.2)	**	28.6 (9.8)	27.3 (11.6)	ns	28.2 (10.7)	22.9 (11.8)	*
Mother’s education												
Primary and lower	25.0 (11.6)	26.3 (13.7)	ns	24.4 (14.6)	18.9 (13.5)	*	32.9 (14.6)	32.1 (10.5)	ns	29.7 (13.3)	27.2 (15.2)	ns
Secondary	28.1 (10.1)	26.7 (10.5)	ns	30.5 (10.1)	27.1 (10.2)	**	29.7 (10.2)	28.4 (10.6)	ns	31.1 (10.4)	28.7 (11.6)	ns
University	26.4 (10.5)	25.0 (10.9)	*	26.3 (11.6)	22.8 (11.7)	**	30.1 (9.1)	28.1 (10.3)	*	28.9 (8.4)	26.0 (9.7)	**
Dating violence experience												
I have never been	25.9 (10.4)	24.5 (11.7)	*	27.2 (12.2)	24.1 (13.1)	**	29.2 (10.3)	27.7 (10.3)	ns	28.5 (9.8)	25.6 (11.4)	*
Yes	28.1 (10.9)	26.5 (10.7)	ns	30.9 (10.2)	27.1 (11.6)	*	30.1 (11.7)	28.8 (12.6)	ns	29.9 (10.2)	28.3 (12.7)	ns
No	28.0 (10.3)	27.5 (10.2)	ns	27.3 (11.7)	23.0 (10.5)	**	30.9 (9.9)	29.4 (9.8)	ns	30.3 (10.0)	27.6 (11.0)	*
Student Social Support Scale												
Low < 232	25.8 (10.2)	24.5 (10.8)	ns	26.4 (11.6)	23.4 (12.7)	**	28.0 (9.3)	27.1 (10.8)	ns	27.3 (8.8)	25.4 (10.1)	ns
Middle 232–276	27.8 (10.8)	26.7 (11.0)	ns	27.5 (11.1)	24.8 (11.0)	**	30.6 (9.4)	28.9 (8.8)	*	28.4 (9.5)	26.0 (11.1)	*
High > 276	27.8 (10.4)	26.9 (11.3)	ns	29.8 (12.2)	24.1 (11.6)	**	32.0 (11.9)	30.1 (11.5)	ns	32.5 (10.7)	29.0 (12.2)	*
Assertiveness												
Low < 82	25.1 (9.8)	25.2 (10.5)	ns	24.6 (10.8)	22.2 (10.7)	*	26.1 (8.8)	26.7 (10.2)	ns	28.1 (9.3)	26.3 (10.2)	ns
Middle 82–92	27.1 (9.9)	25.9 (10.7)	ns	28.3 (11.4)	25.8 (11.9)	*	31.5 (10.1)	29.4 (9.3)	*	30.6 (8.4)	26.5 (12.3)	*
High > 92	28.7 (11.3)	26.5 (11.8)	*	30.2 (12.0)	24.5 (12.1)	**	33.5 (10.9)	30.3 (11.6)	*	30.6 (11.4)	28.2 (11.8)	*
Social Problem-Solving Inventory Revised												
Low < 54	28.3 (10.1)	28.2 (10.4)	ns	27.1 (12.0)	23.5 (11.8)	**	30.8 (9.7)	30.4 (9.9)	ns	31.1 (10.2)	29.0 (10.2)	ns
Middle 54–64	25.4 (9.6)	23.9 (10.7)	*	28.0 (11.6)	25.9 (11.6)	*	28.6 (10.5)	26.1 (10.3)	*	29.6 (10.0)	26.9 (11.6)	ns
High > 64	27.3 (11.4)	25.5 (11.5)	*	28.7 (11.5)	23.5 (11.7)	**	31.3 (10.9)	29.9 (10.9)	ns	28.9 (9.9)	25.8 (12.0)	*
Aggression Questionnaire Refined												
Low < 23	27.1 (10.9)	26.1 (11.3)	ns	27.6 (11.0)	22.1 (11.4)	**	28.8 (12.5)	26.6 (11.9)	ns	29.3 (10.5)	25.1 (12.4)	ns
Middle 23–31	26.2 (10.4)	24.9 (11.5)	ns	27.7 (12.1)	25.2 (11.1)	*	29.2 (9.9)	28.7 (9.1)	ns	29.6 (8.5)	27.5 (9.6)	ns
High > 31	27.6 (10.0)	26.6 (10.2)	ns	28.5 (11.9)	24.9 (12.5)	**	31.7 (9.0)	29.9 (10.3)	ns	30.3 (10.7)	28.7 (11.6)	ns

n.s.: *p*-value> 0.05; *p-value< 0.05; **p-value< 0.001; Student paired t-test

Table 3 shows the variables independently associated with a change in the mean values of BS over time (W2-W1). In girls in W2, there was a significant reduction in the levels of BS in the intervention group compared to the control group (β = − 0.101; *p* = 0.006). This decrease was not observed for boys (*p* = 0.537). For both sexes, the change in mean BS values was associated with the baseline values for BS. Greater baseline values for BS (W1) were associated with a greater reduction in W2. This effect was independent of belonging to the control or intervention group.

**Table 3:** Linear regression for change in subscales

Variable (change in sexism values)	Benevolent sexism (BS)				Hostile sexism (HS)			
	Girls		Boys		Girls		Boys	
	β	***p***-value	β	***p***-value	β	***p***-value	β	***p***-value
Sexism (BS or HS) in baseline	−0.507	0.000	−0.491	0.000	−0.385	0.000	−0.561	0.000
Group (Ref: control)								
Intervention	−0.101	0.006	− 0.029	0.537	0.039	0.319	0.032	0.472
Age	0.077	0.245	0.128	0.105	0.078	0.269	0.091	0.226
Mother’s education (Ref: Primary)								
Secondary	−0.072	0.263	−0.080	0.263	−0.080	0.240	−0.021	0.759
University	−0.085	0.201	−0.127	0.098	−0.086	0.220	−0.099	0.168
Dating violence (I have never been in)								
Yes	0.019	0.617	0.034	0.506	0.042	0.309	0.076	0.111
No	0.029	0.447	0.003	0.946	−0.032	0.433	−0.045	0.353
Student Social Support Scale (Ref: Low < 232)								
Middle 232–276	0.050	0.223	0.008	0.892	0.048	0.272	−0.012	0.825
High > 276	0.074	0.117	0.077	0.213	0.054	0.280	−0.005	0.932
Assertiveness (Ref:Low < 82)								
Middle 82–92	0.023	0.577	−0.030	0.582	−0.052	0.243	0.004	0.943
High > 92	−0.034	0.485	0.014	0.815	−0.006	0.905	0.035	0.522
SPSI-R (Ref: Low < 54)								
Middle 54–64	−0.042	0.291	−0.106	0.047	0.023	0.586	−0.064	0.205
High > 64	−0.076	0.088	−0.055	0.351	0.010	0.828	−0.001	0.982
AQR-12 (Ref: Low < 23)								
Middle 23–31	0.052	0.198	0.104	0.058	0.021	0.630	0.055	0.292
High > 31	0.027	0.545	0.098	0.110	0.032	0.497	0.124	0.034

*β* Standardized beta coefficient; models adjusted by country, *SPSI-R* Social Problem-Solving Inventory Revised, *AQR-12* Aggression Questionnaire Refined, *Ref* Reference

**Fig. 1:**
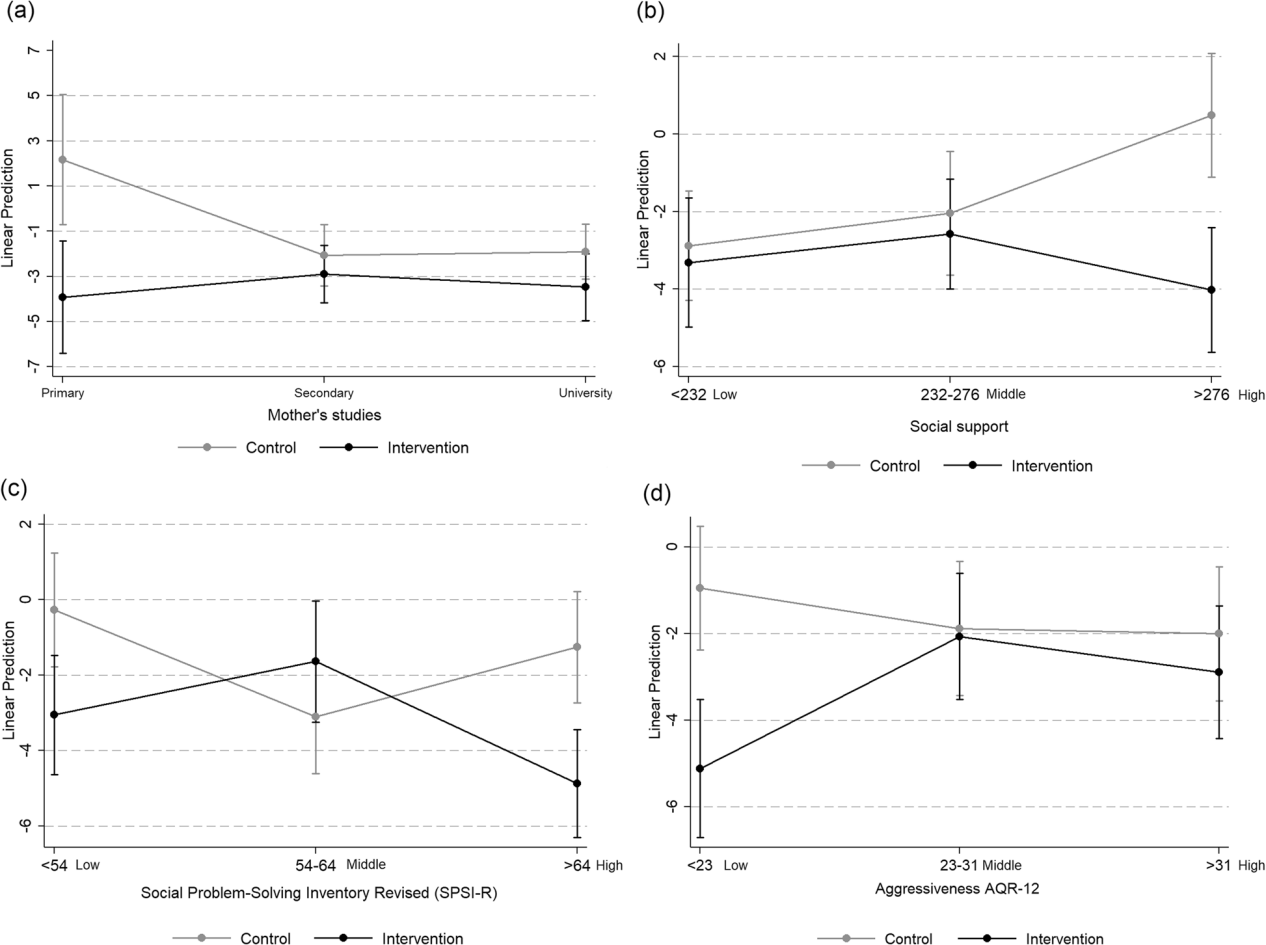
Graphs of significant interactions between the control group and intervention group and the linear regression model variables, benevolent sexism in girls

In the reduction in BS in the subsample of girls, we identified a significant interaction between group (control/intervention) and the following variables: mother’s education (*p* = 0.032), Student Social Support (*p* = 0.009), Social Problem-Solving (*p* = 0.002) and Aggression (*p* = 0.019). In W2 there was a significant reduction in the average values of BS in the intervention group, compared to the control group, in girls with mothers with primary or lower levels of education (Fig. 1a; β = − 0.338; *p* = 0.001), in girls who had a high level of social support at W1 (Fig. 1b; β = − 0.251; *p* < 0.001), a high capacity to resolve social conflicts at W1 (Fig. 1c; β = − 0.201; *p* < 0.001) and low levels of aggressiveness at W1 (Fig. 1d; β = − 0.232, *p* < 0.001). There were no significant interactions in BS in boys,

### Results on hostile sexism

Table 2 shows average values for HS in W1 and W2, by group (control/intervention) and by sex. The effect size was similar in the intervention group (effect size girls: 0.07; effect size boys: 0.03) and in the control group (effect size girls: 0.11; effect size boys: 0.06). Significant decrease was observed in HS among girls in both groups (control/intervention). However, HS remained constant in boys. For girls there was a significant reduction in the levels of HS in some categories of the variables analyzed both in the control and intervention groups. In the intervention group in boys, HS decreased only for those whose mothers had university studies (*p* = 0.032). In the boys in the control group, there was no observed significant change in average levels of HS between W1 and W2.

Table 4 shows the variables independently associated with a change in the mean values of HS over time (W2-W1). There was no significant change in the average levels of HS over time in the control group, compared to the intervention group, for either of the sexes (p_girls_ = 0.319; p_boys_: 0.472). For both sexes, the change in mean HS values was associated with the baseline values for HS. Greater baseline values for HS (W1) were associated with a greater reduction in W2. This effect was independent of belonging to the control or intervention group. There were no significant interactions in HS in both boys and girls.

**Table 4:** Means and standard deviations for hostile sexism by demographic, socioeconomic and violence-related variables at baseline

	Girls						Boys					
	Control			Intervention			Control			Intervention		
	Pre-test	Post-test		Pre-test	Post-test		Pre-test	Post-test		Pre-test	Post-test	
	Mean (SD)	Mean (SD)		Mean (SD)	Mean (SD)		Mean (SD)	Mean (SD)		Mean (SD)	Mean (SD)	
Total	23 (9.7)	21.4 (10.5)	**	22.5 (10.2)	21.5 (11.3)	*	29 (10.1)	28.1 (9.9)	ns	28.7 (10.2)	28.2 (10.4)	ns
Age												
< =13 years	21.3 (10.0)	20.8 (11.6)	ns	20.7 (9.3)	20.0 (9.7)	ns	27.9 (10.1)	27.6 (9.9)	ns	26.6 (10.1)	25.8 (10.2)	ns
14–15 years	23.5 (9.5)	21.3 (10.1)	*	23.9 (9.7)	23.4 (10.8)	ns	29.4 (10.3)	28.4 (9.6)	ns	30.4 (9.2)	31.2 (9.9)	ns
> 15 years	23.7 (9.7)	22.3 (10.2)	ns	22.7 (12.7)	19.3 (14.4)	*	30.0 (9.6)	27.9 (11.2)	ns	31.2 (12.5)	27.8 (10.9)	ns
Mother’s education												
Primary and lower	23.7 (12.4)	23.6 (12.2)	ns	20.8 (12.1)	19.7 (13.6)	ns	29.3 (14.1)	29.5 (8.5)	ns	28.4 (11.3)	27.8 (15.0)	ns
Secondary	23.3 (9.6)	21.5 (9.8)	*	24.5 (9.1)	23.3 (9.8)	ns	28.3 (9.6)	27.0 (9.7)	ns	28.8 (9.9)	30.1 (9.8)	ns
University	22.6 (9.3)	20.9 (10.9)	*	20.8 (10.4)	20.1 (11.7)	ns	29.5 (9.4)	28.3 (10.2)	ns	28.7 (10.2)	27.1 (8.8)	*
Dating violence experience												
I have never been	21.3 (9.4)	20.3 (10.9)	ns	20.8 (10.3)	20.3 (12.1)	ns	28.4 (10.2)	27.4 (10.6)	ns	27.7 (11.0)	27.4 (11.0)	ns
Yes	25.0 (11.4)	22.9 (10.5)	ns	25.2 (10.2)	24.8 (11.2)	ns	30.9 (11.4)	30.8 (10.4)	ns	31.3 (8.9)	31.5 (9.7)	ns
No	24.2 (8.9)	22.1 (9.9)	*	22.6 (9.9)	20.9 (10.5)	*	28.8 (9.4)	27.5 (9.1)	ns	28.7 (10.1)	27.7 (10.2)	ns
Student Social Support Scale												
Low < 232	22.5 (9.8)	20.1 (10.6)	*	22.1 (11.0)	21.1 (11.8)	ns	29.1 (8.9)	28.4 (9.5)	ns	28.7 (9.6)	28.8 (10.5)	ns
Middle 232–276	23.2 (9.6)	22.7 (10.2)	ns	22.4 (9.6)	20.9 (11.2)	ns	30.4 (10.5)	29.7 (9.9)	ns	29.4 (9.2)	27.2 (9.7)	ns
High > 276	23.3 (9.8)	21.8 (10.6)	ns	23.0 (10.2)	22.5 (10.8)	ns	27.5 (10.8)	26.1 (10.1)	ns	28.1 (11.5)	28.8 (11.1)	ns
Assertiveness												
Low < 82	23.4 (9.4)	22.7 (10.1)	ns	21.1 (9.5)	19.9 (10.0)	ns	28.5 (9.5)	28.5 (9.7)	ns	28.0 (9.0)	26.6 (9.9)	ns
Middle 82–92	22.8 (9.0)	21.1 (10.8)	*	22.2 (10.0)	20.3 (10.6)	*	29.9 (9.2)	27.9 (9.8)	ns	30.6 (9.6)	29.1 (9.8)	ns
High > 92	22.6 (10.7)	20.5 (10.6)	*	23.8 (10.8)	23.7 (12.3)	ns	28.8 (11.6)	27.6 (10.4)	ns	28.1 (11.4)	29.1 (11.2)	ns
Social Problem-Solving Inventory Revised												
Low < 54	24.8 (9.9)	23.3 (10.8)	ns	24.2 (11.2)	21.7 (12.2)	*	31.8 (9.2)	30.1 (9.4)	ns	30.2 (9.5)	29.7 (9.4)	ns
Middle 54–64	23.6 (9.1)	21.9 (9.9)	*	23.2 (9.0)	22.7 (9.4)	ns	28.3 (10.4)	26.8 (10.4)	ns	29.0 (10.3)	27.3 (11.9)	ns
High > 64	20.6 (9.8)	19.2 (10.5)	ns	20.6 (10.0)	20.5 (11.7)	ns	26.6 (10.1)	27.1 (9.6)	ns	27.3 (10.6)	27.8 (9.8)	ns
Aggression Questionnaire Refined												
Low < 23	21.7 (9.9)	20.8 (9.7)	ns	21.0 (9.6)	19.5 (11.4)	ns	26.4 (10.9)	26.6 (10.9)	ns	26.2 (11.1)	25.3 (11.5)	ns
Middle 23–31	21.7 (9.1)	20.2 (11.3)	ns	21.9 (9.9)	21.0 (10.6)	ns	26.2 (9.4)	24.5 (9.2)	ns	29.0 (9.0)	29.6 (8.4)	ns
High > 31	25.5 (9.6)	23.2 (10.4)	*	24.7 (10.8)	24.0 (11.5)	ns	32.6 (8.9)	31.4 (8.7)	ns	30.7 (9.9)	29.8 (10.4)	ns

n.s.: ***p*-**value> 0.05; *p-value< 0.05; **p-value< 0.001; Student paired t-test

## Discussion

### Main results

The present study shows three main results: a) during the W2-period there was a decrease in BS (both sexes) and in HS (in girls), both in the control group and the intervention group; b) the decrease in BS in girls –and not in boys- was significantly greater in the intervention group than in the control group; c) the intervention decreased BS in girls whose mothers had a low level of education and in those with high levels of social support, high capacity for conflict resolution and low levels of aggressiveness. In boys, the intervention did not produce significant changes in the average levels of BS or HS.

### Possible explanations

The decrease observed in sexism W2 period, both in the cases and in the controls, could show that the act of responding to the questionnaire itself has an effect on sexism levels. The study published by Montañés [43] showed how response to the Ambivalent Sexism Inventory modified the self-perceptions of the interviewees regarding their experiences with inter-partner violence. Interviewees recognized greater exposure to inter-partner violence when they had responded to the ASI than if the information had been collected in reverse order (exposure to the inter-partner violence-ASI questionnaire-). The authors argued that the questionnaire instrument itself could facilitate the recognition of inter-partner violence. In our study this fact is more visible for BS. This is a subtle form of sexism that is, on occasions, difficult to recognize, given that it is culturally incorporated into positive behaviors. It is possible that responding to the questionnaire acts as an external stimulus that permits the participants to become conscious of the attitudes and values that underlie those behaviors and to take a more critical position in terms of rejecting them. Although the time between W1 and W2 was 6 months, it is possible that this change could be due to an age effect, given that BS seems to present an U-shaped trajectory for women across the lifespan and a positive linear trajectory in men [4, 44]. It could also be due to sociocultural and contextual influences outside of the intervention (publicity campaigns, participation in extracurricular activities, etc.) that both the cases and controls could be exposed to. On the other hand, we cannot rule out the presence of social desirability bias.

In terms of our second result, we found that the Lights4Violence intervention promotes a significant decrease in BS among girls. As far as we know, there are few interventions with adolescents whose main result is a decrease in sexism [45, 46]. However, there is evidence that allows us to highlight the reach of these results. Both HS and BS have been identified as predictors of dating violence in adolescents [47]. Women with BS attitudes, beliefs and behaviors are more likely to maintain traditional gender roles [48], which is an attitude that is linked to acceptance of IPV [49, 50]. Greater BS among young girls has been associated with a greater number of sexual partners in adolescence [8], greater idealized myth of romantic love, and greater attraction by partners with BS [37] among others. It is for this reason that implementing interventions that reduce BS in girls could promote egalitarian relationships in adolescence. In this sense, given that sexism places men and boys in a position of domination and power, it is necessary to identify interventions that decrease sexism among men and boys, thus supporting equality and diminishing the need to promote the rejection of sexism among women. The decrease in the mean values of BS and HS in W2 was associated with baseline levels of sexism. When these were high, the reduction was greater. This effect was independent of group (intervention/control) and sex. Finally, our results show a decrease in BS among girls with mothers with low levels of education and in those with high levels of social support, high capacity for conflict resolution and low aggressiveness. This decrease in BS in girls with lower socioeconomic levels at home - whose mothers have lower education levels - is important as it could indicate that the Lights4Violence intervention promotes equity among young people. A recent systematic review published by Tinner [51] shows that there are very few public health interventions with young people that allow for analyzing the effect on different social strata. Thus, if these interventions are not being received among the most disfavored groups, they could be contributing to an increase in health inequalities.

In terms of the decrease in BS in young people with greater capacities and social support, it is possible that it is due to the fact that these characteristics predispose young people to a more egalitarian change in attitudes. In this sense studies by Ferragut 2013 show how positive psychological values are inversely related with sexist attitudes [52]. Specifically, among adolescent girls there is lower sexism among those with greater empathy and understanding of the social reality. Leaper’s studies support this idea and affirm that learning about feminist theory could provide to girls a conceptual model that allows them to become more conscious and position themselves against sexist attitudes [53]. The greater permeability of girls to theories that promote equality between men and women could explain the decrease in BS observed in girls. In fact, the intervention did not produce a substantial change in the average levels of sexism in boys. Given that the concept of sexism makes reference to attitudes, beliefs and behaviors that emphasize the inferiority of women to men based on their sex, the response prompted by questioning of these attitudes through educational interventions could be different between the sexes [37]. It is possible that boys are less receptive to change, and that in questioning the concept of hegemonic masculinity, boys could perceive a loss of privileges. In this sense, the studies carried out by Becker and Swim (2011) [54] show that in men it is not enough to increase their awareness of sexist behavior to promote a change. Changes in sexist attitudes in men occur when empathy towards women who suffer from sexist behaviors and attitudes increases. In our work, we could not analyze this hypothesis because empathy is evaluated in a generic way -not toward women who suffer sexism-, and it was measured in the W1 but not in the W2, so that we could not monitor the change in empathy during the intervention.

It is important to integrate contents to help boys reflect on gender roles and the meaning attributed to hegemonic masculinity in these types of interventions. In this sense a gender transformative approach is promising for preventing risky health behaviors and violence, since it works to foster equitable attitudes, behaviors and community structures that support women and men and help them to break with gender stereotypes [55].

This work should be interpreted taking into account certain limitations. The study is based on a non-probabilistic sample. Schools were selected for their feasibility. The distribution of the schools in the intervention and control groups was not carried out randomly. Even so, the differences identified between the intervention and control groups were introduced into statistical models to avoid spurious associations. Social desirability bias may be present when information is collected on socially committed topics. This bias is minimized when the information is self-completed online using anonymous questionnaires. If it existed, it would be present both in the baseline questionnaire (W1) and in the follow-up questionnaire (W2). If it is present, a decrease in sexism would be observed in the intervention group in the W2, giving rise to an overestimation of the effect of the intervention. This greater decrease in sexism in the intervention group compared to the control group is not observed in boys, which is where the social desirability bias could be present. The time of follow-up of the participants after the intervention was limited in time. We do not know whether the effect of the intervention on sexism can be maintained over time, and in the same way, it is possible that the intervention has an effect on sexism over the long-term that we cannot observe in this study. In our study, because it was not considered culturally appropriate in some of the countries participating, we did not include the gender variable nor the sexual orientation variable. Therefore, we could not estimate the impact of gender identity and sexual orientation on the intervention results on sexism. Given the association of these variables with IPV, it is possible that they were also associated with sexism and with intervention results. Despite this, teaching of sexual and gender diversity was taken into account in the implementation of the intervention. Ethnicity was measured in the study, but the low percentage of people born abroad or with parents born abroad, did not allow for us to include it in the analysis. Thus we could not carry out analysis that included the intersectionality framework.

## Conclusions

This study suggests that it is possible to carry out interventions that promote a decrease in sexism in young women. There is a need to create spaces for reflection that allow for integrating young men into these changes towards more egalitarian relationships. Social support and encouraging social capacities could facilitate this decrease in sexism and thus the promotion of positive interpersonal relationships.

## Data Availability

Data will be available upon reasonable request from the corresponding author. The data are not publicly available due to privacy or ethical restrictions.

## Abbreviations

BS: Benevolent sexism
HS: Hostile sexism
IPV: Intimate partner violence
ASI: Ambivalent Sexism Inventory
SSSS: Student Social Support Scale
AISQ: Assertive Interpersonal Schema Questionnaire
SPSI-R: Social Problem-Solving Inventory-Revised Scale
AQR-12: Aggression Questionnaire
W1: Wave 1
W2: Wave 2
