# Correction: Effect of the Lights4Violence intervention on the sexism of adolescents in European countries

**DOI:** 10.1186/s12889-022-13360-0

**Published:** 2022-05-11

**Authors:** Belén Sanz-Barbero, Alba Ayala, Francesca Ieracitano, Carmen Rodríguez-Blázquez, Nicola Bowes, Karen De Claire, Veronica Mocanu, Dana-Teodora Anton-Paduraru, Miriam Sánchez-SanSegundo, Natalia Albaladejo-Blázquez, Ana Sofa Antunes das Neves, Ana Sofa da Silva Queirós, Barbara Jankowiak, Katarzyna Waszyńska, Carmen Vives-Cases

**Affiliations:** 1 National School of Public Health, Carlos III Institute of Health, Avda. Monforte de Lemos, 5, –28029 Madrid, Spain; 2 CIBER of Epidemiology and Public Health (CIBERESP), Madrid, Spain; 3 Research Network on Health Services for Chronic Diseases (REDISSEC), Madrid, Spain; 4 Department of Human Studies-Communication, Education and Psychology - LUMSA University of Rome, Rome, Italy; 5 National Center of Epidemiology, Carlos III Institute of Health, Madrid, Spain; 6 CIBER in Neurodegenerative Diseases (CIBERNED), Madrid, Spain; 7 Department of Applied Psychology, Cardif Metropolitan University, Wales, UK; 8 Mother and Child Medicine Deparment, Gr.T. Popa University of Medicine and Pharmacy, Iasi, Romania; 9 Faculty of Health Science, University of Alicante, Alicante, Spain; 10 University of Maia and CIEG/ISCSP-ULisboa, Porto, Portugal; 11 University Institute of Maia – ISMAI, Maia, Portugal; 12 Faculty of Educational Studies, Adam Mickiewicz University, Poznań, Poland

**Correction to: BMC Public Health 22, 547 (2022)**

**https://doi.org/10.1186/s12889-022-12925-3**

The original publication of this article [1] contained 2 swapped table citations. The incorrect and correct information is shown below. The original article has been updated.

**Incorrect**

- Table 2 shows average values for HS in W1 and W2, by group (control/intervention) and by sex
- Table 4 shows the variables independently associated with a change in the mean values of HS over time (W2-W1).

**Correct**

- Table 4 shows average values for HS in W1 and W2, by group (control/intervention) and by sex
- Table 3 shows the variables independently associated with a change in the mean values of HS over time (W2-W1).

## References

[1] Sanz-Barbero B (2022). Effect of the Lights4Violence intervention on the sexism of adolescents in European countries. BMC Public Health.

